# Polymorphisms in voltage-gated sodium channel gene and susceptibility of *Aedes albopictus* to insecticides in three districts of northern West Bengal, India

**DOI:** 10.1371/journal.pntd.0006192

**Published:** 2018-01-08

**Authors:** Moytrey Chatterjee, Sudeep Ballav, Ardhendu K. Maji, Nandita Basu, Biplab Chandra Sarkar, Pabitra Saha

**Affiliations:** 1 Department of Microbiology, Calcutta School of Tropical Medicine, Kolkata, West Bengal, India; 2 Director, Calcutta School of Tropical Medicine, Kolkata, West Bengal, India; 3 Department of Zoology, A. P. C. Roy Govt. College, Himachal Bihar, Matigara, Siliguri, West Bengal, India; University of Texas at Austin, UNITED STATES

## Abstract

**Background:**

The control and prevention of dengue largely depends on vector control measures, environmental management, and personal protection. Dengue control programmes are facing great challenges due to development of insecticide resistance among vector mosquitoes. Information on susceptibility status to different insecticides is important for national programmes to formulate vector control strategies.

**Methods:**

We have studied the larval susceptibility of *Aedes albopictus* to temephos and adult susceptibility to 4% DDT, 0.05% deltamethrin, and 5% malathion as per WHO protocols in the northern districts of West Bengal. Polymorphisms in the *VGSC* gene were studied by direct sequencing of PCR products.

**Results:**

The *Ae*. *albopictus* larval population showed sensitive [Resistance Ratio (RR_99_)<3] to moderate levels of resistance (5<RR_99_>10) to temephos at different study sites. Adult bioassay results revealed that *Ae*. *albopictus* was highly resistant to DDT [Corrected Mortality (CM) < 90%] in all the study sites and susceptible to deltamethrin and malathion (CM > 98%), except in Dhupguri where a low level of resistance to deltamethrin (CM = 96.25%) was recorded. None of the six important *kdr* mutations (S953P, I975M/V, L978, V980G, F1474C, D1703Y) were found in the *VGSC* of studied mosquitoes, but we identified 11 synonymous and 1 non-synonymous mutation in the *VGSC* gene.

**Conclusion:**

The higher susceptibility level to deltamethrin and malathion, along with the absence of important *kdr* mutations indicates that these two insecticides are still effective against *Ae*. *albopictus* in the study areas. The susceptibility status of temephos should be monitored closely as low to moderate levels of resistance were observed in few sites. A similar study is recommended for monitoring and early detection of insecticide resistance in other parts of the country.

## Author summary

Dengue is one of the most important arboviral infections in India, and transmitted by *Aedes* mosquitoes. Control of dengue largely depends on effective vector control measures as no specific drugs or vaccines are available, to date. The knowledge of insecticide susceptibility status for local vector mosquitoes is essential for the formulation of effective vector control measures. Therefore, regular monitoring of insecticide susceptibility is essential for the early detection of resistance. In the present study, sensitive to moderate levels of resistance to temephos were recorded among the *Aedes albopictus* larval populations of different study sites. Adult *Ae*. *albopictus* were highly resistant to DDT and susceptible to deltamethrin and malathion in all the study sites, except one site where a low level of resistance to deltamethrin was observed. By sequencing the *VGSC* gene we observed that the studied *Ae*. *albopictus* population does not contain any of the important *kdr* mutations which are associated with DDT and pyrethroid resistance in other insects. We found only one non-synonymous mutation at codon S1485**L,** but its role in pyrethroid resistance is yet to be established.

## Introduction

Dengue is a mosquito-borne flavi-viral disease and a major public health problem in more than 120 countries [1, 2]. In recent years, dengue transmission has increased predominantly in urban, semi-urban areas and has even extended to the rural areas, becoming a major public health concern globally. A recent estimate showed 390 million new dengue infections throughout the world, of which, 96 million cases manifested the severe form of the disease [2] and almost half of the world’s population are at risk of dengue infection [3]. In India, dengue is spreading into new areas and emerging as a major public health problem. In 2016, a total of 129166 dengue cases and 245 deaths were reported from India, of which 22865 cases and 45 deaths were reported from West Bengal [4]. *Aedes aegypti* and *Aedes albopictus* are the vectors of dengue along with three other important human viral diseases: yellow fever, chikungunya, and Zika. No effective vaccine against dengue is available to date. Vector control and personal protection from mosquito bites are suggested to reduce its transmission. For proper formulation and implementation of vector control strategies, thorough information about vector species distribution and their susceptibility to available insecticidal agents are necessary [5].

Four different classes of insecticides are in use as adulticides against *Aedes* mosquitoes: organophosphates, pyrethroids, organochlorines, and carbamates [6, 7]. Among these, pyrethroids and organophosphates are widely used throughout the world [8, 9, 10]. Pyrethroids are used as indoor residual treatment and impregnation of bed nets whereas organophosphates are used as larvicides and space treatments [6]. The National Vector Borne Disease Control Programme (NVBDCP) of India recommends different insecticides for vector management, such as temephos (50 EC) as a larvicide, DDT and synthetic pyrethroids (recently introduced) for indoor residual spray (IRS), deltamethrin (pyrethroid) for impregnation of bed nets, and malathion for ultra low volume (ULV) spray. In India, *Aedes* mosquito control is mainly based on anti-larval measures and the use of insecticides by space spraying of pyrethrum and fogging of malathion during a disease outbreak to kill adults. The development and spread of resistance by the vector mosquitoes against all available insecticides is a great challenge to prevent the transmission of mosquito-borne diseases. *Ae*. *albopictus and Ae*. *aegypti* showed resistance to DDT [11, 12, 13], but were susceptible to malathion and deltamethrin [11, 12, 14, 15] in different parts of India. Pyrethroids are synthetic analogues of naturally occurring pyrethrum from the extracts of the *Chrysanthemum* flower and represent the most widely used insecticide against insect vectors [16]. Unfortunately, pyrethroid efficacy is being threatened due to rapid development of resistance by the vector mosquitoes [8, 17]. The World Health Organisation (WHO) formulated standard diagnostic bioassay test kits to monitor the susceptibility of mosquitoes against different insecticides [18].

Exposure to pyrethroids and DDT results in “knockdown” (i.e., rapid paralysis) due to prolonged-activation of sodium channels. Pyrethroids and organochlorines cause overstimulation of the mosquito nervous system by repeated action potentials form the opening of the sodium channel [19, 20, 21]. Knockdown resistance (*kdr*) is the major mechanism of pyrethroid resistance, caused by mutations in the voltage-gated sodium channel gene (*VGSC* gene) [22, 23]. In insects, the voltage-gated sodium channel is an integral transmembrane protein which is composed of four homologous domains (I-IV). Each domain consists of six subunits (S1-S6) which are connected by loops. The segments S5, S6, and the P-loop between them form a central aqueous pore, and the S1-S4 segments of each domain unite to form four independent voltage-sensitive domains [24, 25]. Insects have only one functional sodium channel gene [19]. There are two receptor sites in the four-domain sodium channel for simultaneous binding of pyrethroids [26].So far, ten different mutations at eight codons comprising fifteen haplotypes have been reported in *Ae*. *aegypti*. The frequency of these mutations varies geographically [27, 28] but such reports from India are very rare.

Periodical monitoring of insecticide resistance among the prevailing vector population in a given geographical region will be helpful to formulate vector control strategies by the NVBDCP. The present work was designed to study the susceptibility status of *Ae*. *albopictus* to temephos, DDT, deltamethrin, and malathion, as well as polymorphisms in the *VGSC* gene in dengue endemic areas of northern West Bengal.

## Materials and methods

### Study sites

**Fig 1:**
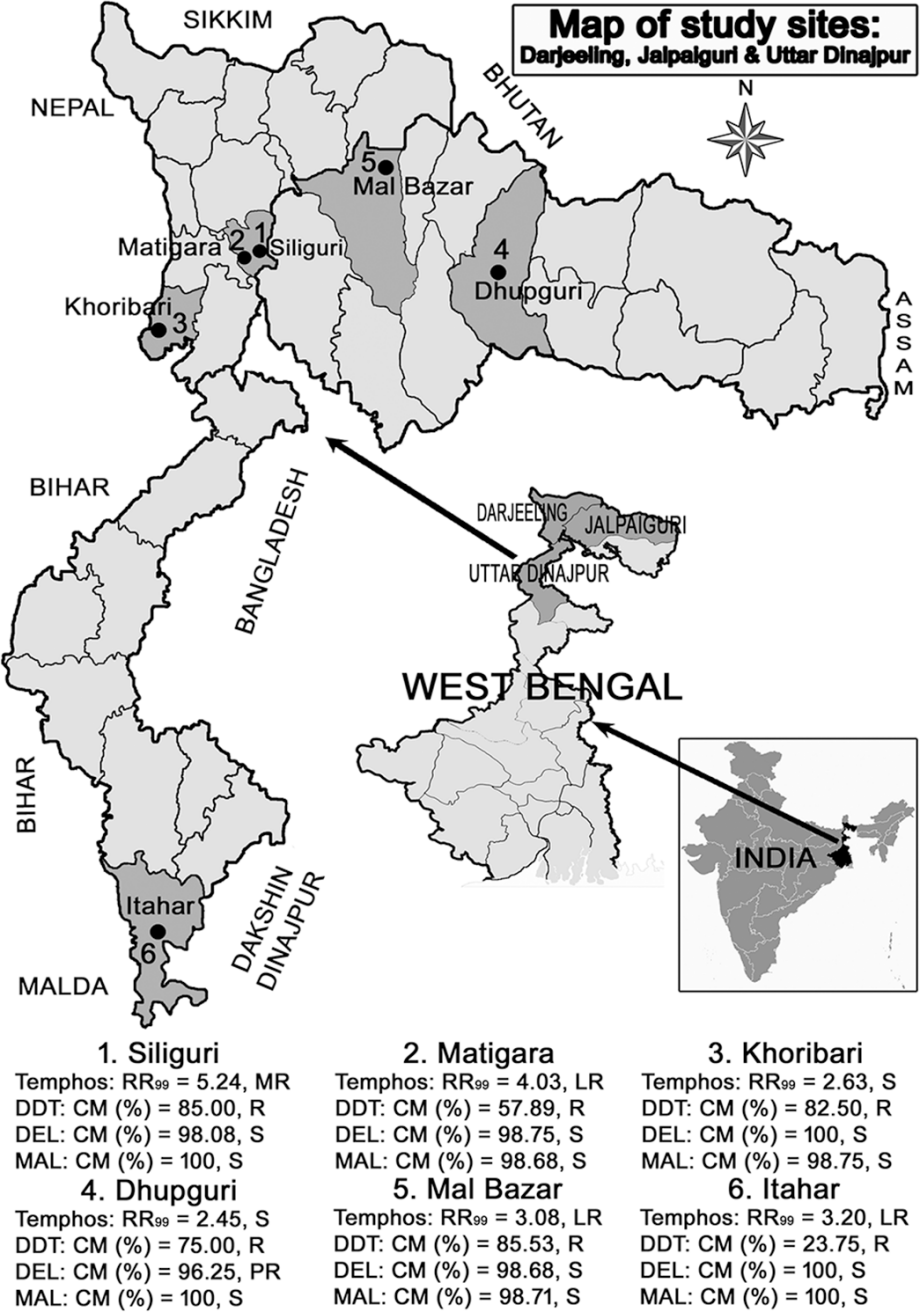
Map showing the study sites.

This study was carried out in one municipality and two blocks of Darjeeling, two blocks of Jalpaiguri, and one block of Uttar Dinajpur districts of West Bengal during June 2016 to September 2016. The study locations were Siliguri Municipal Corporation (SMC), Matigara, and Khoribari of the Darjeeling district; Malbazar, Dhupguri of the Jalpaiguri district, and the Itahar block of Uttar Dinajpur. Most of the study sites were sub-urban except Siliguri Municipal Corporation (urban) and Khoribari (rural) (Fig 1).

### Mosquito collection, rearing and identification

The aquatic stages (larvae and pupae) of *Aedes* sp. were collected from the seventeen localities of three districts. For each collection site, larvae and pupae were collected from domestic, peri-domestic and natural breeding places. The collected immature stages of mosquitoes were stored in plastic containers containing water from the same breeding habitat and transferred to the laboratory. In the laboratory, the wild caught mosquito larvae and pupae were transferred into a larvae rearing tray along with water collected from the field and supplied with food for ornamental fishes available in the local market along with yeast. The mosquito larvae and pupae were reared to the adult stages in the laboratory under controlled conditions (temperature 25°C ± 2°C; relative humidity 80% ± 10%). After emergence, the adults were identified by using the standard identification keys of Barraud, 1934 [29] and Tyagi *et al*., 2012 [30]. The identified *Ae*. *albopictus* were allowed to breed under laboratory conditions. The larvae and adults of the F1 generation were used for larval and adult insecticide bioassays.

### Larval susceptibility tests

Susceptibility of larvae to temephos (50EC; Nitapol Industries Pvt Ltd., Kolkata) was estimated using the standard WHO bioassay protocol [31]. The stock temephos solution of 1 ppm concentration and other subsequent dilutions were prepared in 95% ethanol and stored at +4°C for use in the susceptibility bioassay. Bioassays were conducted using 20–25 third instar to early fourth instar larvae (wild caught strain and laboratory strain) in disposable paper cups filled with the required concentration of insecticide solution and double distilled water at room temperature (25°C ± 2°C). Eight different concentrations (0.0005, 0.001, 0.005, 0.01, 0.05, 0.1, 0.5 and 1.0 ppm) were used as per WHO recommendation [32, 33] and each experiment was replicated at least three times. Each set of the bioassay was accompanied by two sets of controls (equal concentration of 95% ethanol). Larval mortality was recorded after 24 h of exposure. The larvae that were motionless or convulsive upon a sharp stimulation were counted as dead [31]. Larval mortality was determined by dividing the number of dead larvae by the total number tested. A test was considered as invalid if pupation rate was greater than 10%, or mortality rate in the control was greater than 20% [31]. The degree of resistance was determined by the resistance ratio (RR_99_), which is calculated by comparing the lethal concentration (LC_99_) value for a population with the LC_99_ value for the insecticide for a laboratory colony. The RR_99_ ≤3 was considered as susceptible, and 3 < RR_99_ ≤ 5 as low resistance, 5 < RR_99_ ≤ 10 as moderate resistance, and RR_99_> 10 as high resistance [34].

### Adult susceptibility bioassay

Two to three day old laboratory emerged unfed female *Ae*. *albopictus* mosquitoes were used for the insecticide susceptibility bioassay as per WHO protocol [18]. The tested insecticides were 4% DDT, 0.05% deltamethrin, and 5% malathion. The insecticide-impregnated papers were procured from the Vector Control Research Unit (VCRU), Universiti Sains Malaysia, Malaysia. Five different holding tubes were used for each set of the experiment of which four were a test and one was a control. In each holding tube,15–20 adult female mosquitoes were kept for one hour. After one hour of holding, mosquitoes from four tubes marked as test were exposed to insecticide-impregnated papers. The control tests were performed using silicone oil, olive oil, and risella oil pre-impregnated papers for deltamethrin, malathion, and DDT, respectively. Mosquitoes were allowed in the exposure tube for one hour and cumulative knock down was recorded after 10, 15, 20, 30, 40, 50, and 60 minutes. After 60 minutes of exposure, the mosquitoes were transferred to holding tubes and fed on a 5% sucrose solution for the next 24 h. Mortality was scored after 24 h to determine the susceptibility status as per WHO recommendation [18]. Mosquitoes were considered dead if they were motionless, when they were mechanically stimulated, following the method of Gonzalez Audino [35]. The live and dead mosquitoes obtained from the adult bioassays were stored at -20°C and used for molecular biological assays.

### DNA isolation and *kdr* mutation detection

Genomic DNA was isolated from both live and dead mosquitoes (individually) by using the DNeasy Blood & Tissue Kit (Qiagen, Germany), as per the manufacturer’s instructions. Before initiation of DNA isolation, the wings of the mosquitoes were removed and the remaining part of the mosquito was carefully homogenised by a Tissue Ruptor (Qiagen, Germany). Extracted DNA was stored at -20°C until further study.

PCR was done using three different primer pairs targeting six amino acid loci (S953P, I975M/V, L978, V980G of domain II, F1474C of domain III and D1703Y of domain IV) of the voltage-gated sodium channel gene (*VGSC*) of *Ae*. *albopictus*, which is responsible for knockdown resistance (*kdr*). The details of primers and PCR conditions are given in Table 1 as described earlier by Kasai *et al*., 2011 [36].

**Table 1:** Primer and PCR conditions used for amplification and sequencing of *VGCS* gene of *Aedes albopictus* (Kasai *et al*., 2011).

Domains	Primer name	PCR Primers (5'-3')	PCR condition	Sequencing primers (5'-3')
II	aegSCF20	GACAATGTGGATCGCTTCCC	Initial denaturation at 94°C for 3 min, 35 cycles each of 94°C for 15 s, 55°C for 30 s, and 72°C for 30 s, followed by a final elongation step at 72°C for 10 min	**aegSCF3:** GTGGAACTTCACCGACTTCA
	aegSCR21	GCAATCTGGCTTGTTAACTTG		**aegSCR22:** TTCACGAACTTGAGCGCGTTG
III	aegSCF7	GAGAACTCGCCGATGAACTT		**aegSCR8:** TAGCTTTCAGCGGCTTCTTC
	aegSCR7	GACGACGAAATCGAACAGGT		
IV	albSCF6	TCGAGAAGTACTTCGTGTCG		**albSCF7:** AGGTATCCGAACGTTGCTGT
	albSCR8	AACAGCAGGATCATGCTCTG		

The quality of PCR products was ascertained by 2% agarose gel electrophoresis following ethidium bromide stain. The PCR product was gel purified using the Qiagen gel extraction kit (Qiagen, Germany) and sequencing was outsourced to Chromous Biotech, Bangalore. Four different primers i.e., aegSCF3, aegSCR22 (forward and reverse primer for domain II), aegSCR8 (reverse primer for domain III), and albSCF7 (forward primer for domain IV) were used for sequencing of the PCR products.

### Analysis of sequence

In the present study, we numbered the codons of the *VGSC* gene according to the sequence of *Ae*. *albopictus*. The sequences were analysed using the software BioEdit Sequence Alignment Editor version 7.0.9.0. The sequences were aligned with the reference sequence for *Ae*. *albopictus* (GenBank accession no. AY663384.1), using an online multiple sequence alignment tool.

### Ethics statement

Before initiation of the work, the objectives of the study were explained to the local population of each study site. Permission was taken from the owners of private houses/lands before collection of immature stages of mosquito. The study did not involve any endangered and protected species. Mosquitoes were maintained under optimal conditions such as temperature, humidity, and adequate food supply in the laboratory. The study protocol was approved by the Institutional Ethics Committee of Calcutta School of Tropical Medicine, Kolkata.

### Data analysis

Larval bioassay data were analyzed using Log dose probit (Ldp) Line computer software (Ehabsoft, Cairo Egypt; available at: http://www.ehabsoft.com/ldpline) according to the Finney’s method [37]. Chi-squared (χ^2^) test was used to estimate the goodness of fit, while linear regression was used to evaluate the data linearity. Lethal concentrations (LC_10_, LC_50,_ and LC_99_) along with the slope were estimated at 95% confidence intervals (CI). For adult bioassays, observed mortality was calculated by the formula: observed mortality (%) = (Total no. of dead mosquitoes / Total mosquitoes exposed) x 100. The observed mortality was corrected using Abbott’s formula when the mortality rate of control was within 5% - 20%. Corrected Mortality (CM) (%) = [(% of observed mortality—% of control mortality) / (100 - % of control mortality)] x 100. For adult bioassays, resistant/susceptibility status was defined according to WHO recommendations [18]. Mosquitoes were considered susceptible (S) if the corrected mortality (CM) rate was greater than 98% and resistant (R) if mortality rate was less than 90%. Mortality rate between 90–98% was considered as possible resistance (PR) and needs verification by alternative methods like enzyme bioassay and molecular marker studies [18]. The cumulative knock down rates (KDR) were calculated by observing the number of knocked down mosquitoes after 10, 15, 20, 30, 40, 50 and 60 minutes during the hour-long exposure period. Knockdown time (KDT_10_, KDT_50,_ and KDT_95_) is the time required for knockdown of a particular proportion of mosquitoes following exposure to any insecticide. KDTs were determined using Log dose probit (Ldp) Line computer software (Ehabsoft, Cairo Egypt; available at: http://www.ehabsoft.com/ldpline) programme according to the Finney’s method [37].

## Results

### Demography of the study area

The study was conducted in one municipality and 5 different blocks of 3 districts in the northern part of West Bengal during June 2016 –September 2016. The study sites of Dhupguri and Itahar blocks were surrounded by paddy fields, whereas the presence of both paddy fields and tea gardens were characteristic of the remaining study sites except Siliguri Municipality Corporation (SMC) and Matigara. Most of the study sites were suburban in nature except the Siliguri municipality area (Urban) and Khoribari (rural) (Fig 1). Storage water tanks, discarded tyres, tree holes, construction sites, flower pots, plastic cups, coconut shells, and discarded containers were the different seasonal breeding sites found in the study area. The climatic conditions of all study sites were humid and sub-tropical in nature and the temperature varies from 8°C in winter to 40°C in summer.

### Larval susceptibility status

The summary of larval bioassay results is presented in Table 2. The LC_10_, LC_50,_ and LC_99_ values of different study sites did not follow a normal distribution for mortality to the log dose (χ^2^ ≥ 16.08; p ≤ 0.01). The LC_50_ values ranged from 0.0009 to 0.0015 mg/L and LC_99_ from 0.1565 to 0.3343 mg/L. The calculated RR_50_ and RR_99_ values in different study sites were ranged from 1.0 to 2.5 and 2.45 to 5.24, respectively.

**Table 2:** Temephos susceptibility status of *Aedes albopictus* in three districts of West Bengal.

Values	Study sites					
	Darjeeling			Jalpaiguri		Uttar Dinajpur
	Siliguri (n = 480)	Matigara (n = 480)	Khoribari (n = 480)	Dhupguri (n = 480)	Malbazar (n = 480)	Itahar (n = 480)
**LC**_**10**_ (lower limit–upper limit) [mg/L]	0.0001 (0–0.0001)	0.0001 (0–0.0001)	0.0001 (0–0.0001)	0.0001 (0–0.0001)	0.0001 (0–0.0001)	0.0001 (0–0.0001)
**LC**_**50**_ (lower limit–upper limit) [mg/L]	0.0015 (0.0005–0.0028)	0.0009 (0.0002–0.0018)	0.001 (0.0001–0.0019)	0.0006 (0.0001–0.0011)	0.001 (0.0002–0.0021)	0.0009 (0.0002–0.0019)
**LC**_**99**_ (lower limit–upper limit) [mg/L]	0.3343 (0.1699–2.8605)	0.2574 (0.1616–3.7848)	0.1678 (0.2453–19.1891)	0.1565 (0.1838–16.7816)	0.1963 (0.1414–4.5084)	0.2043 (0.1763–8.025)
**X**^**2**^ **(p)**	16.08 (0.01)	23.97 (0.0005)	34.72 (<0.0001)	27.93 (0.0001)	23.59 (0.0006)	26.68 (0.0002)
**Slope**	0.99 ± 0.09	0.95 ±0.09	1.03 ± 0.11	0.97 ± 0.11	1.02 ± 0.11	0.99 ± 0.11
**R**	0.95	0.92	0.91	0.91	0.93	0.92
**G**	0.14	0.21	0.39	0.38	0.26	0.29
**RR**_**50**_**/RR**_99_*	2.5 / 5.24	1.5 / 4.03	1.67 / 2.63	1.0 / 2.45	1.67 / 3.08	1.5 / 3.20
**Status**^**#**^	MR	LR	S	S	LR	LR

**n** = number; **LC**_**10**_**/LC**_**50**_**/LC**_**99**_ = lethal concentration 10%/50%/99%, **RR** = resistance ratio, g = ‘g’ is a factor used for fiducial limit calculations

* The LC_50_ and LC_99_ values of laboratory strain was 0.0006mg/L and 0.0638mg/L, respectively

#Classification adapted from Mazzari and Georghiou (1995): S = Susceptible (RR < 3), LR = Low Resistance (3 < RR < 5), MR = Moderate Resistance (5 < RR < 10), HR = High Resistance (>10).

### Adult susceptibility status

The results of the adult susceptibility bioassay for *Ae*. *albopictus* are given in Table 3. After 24 hours of exposure, the corrected mortality rates for 4% DDT were 23.75% to 85.53% in different study sites. The obtained mortality rates were well below the WHO recommended 90% mortality rate for resistance. So, results suggested that the *Ae*. *albopictus* population of the study areas was highly resistant to DDT. In all of the study sites, the corrected mortality rate for 0.05% deltamethrin ranged from 98.08% to 100%, except in Dhupguri where the corrected mortality was 96.25%. So, *Ae*. *albopictus* population of all the study sites was susceptible to deltamethrin except Dhupguri. The corrected mortality rate for 5% malathion was >98% in all the study sites indicating susceptibility to malathion.

**Table 3:** Insecticide susceptibility status of *Aedes albopictus* against 4% DDT, 0.05% deltamethrin, and 5% malathion in West Bengal.

Insecticides	Districts	Blocks	Mosquito exposed		Mosquito died		Observed Mortality (%)		CM (%)	KDT_10_ (min.) [95% CI]	KDT_50_ (min.) [95% CI]	KDT_95_ (min.) [95% CI]	χ^2^ (p)	Slope	Status^#^
			T*	C*	T*	C*	T*	C*							
**4% DDT**	Darjeeling	Siliguri	160	40	136	0	85.00	0	**85.00**	12.82 [9.05–14.75]	28.59 [23.6–34.39]	80.10 [69.57–119.04]	**19.59 (0.002)**	**3.68 (± 0.2)**	**R**
		Matigara	160	40	96	2	60.00	5.00	**57.89**	11.98 [9.45–14.24]	42.17 [37.90–47.91]	212.11 [155.71–329.21]	**3.24 (0.7)**	**2.34 (± 0.2)**	**R**
		Khoribari	160	40	132	0	82.50	0	**82.50**	13.26 [11.47–14.89]	30.36 [28.31–32.57]	87.91 [76.28–105.28]	**7.53 (0.2)**	**3.56 (± 0.2)**	**R**
	Jalpaiguri	Dhupguri	160	40	122	2	76.25	5.00	**75.00**	17.39 [11.08–20.41]	51.39 [43.11–73.09]	206.49 [172.26–538.03]	**15.83 (0.007)**	**2.72 (± 0.3)**	**R**
		Malbazar	160	40	138	2	86.25	5.00	**85.53**	7.65 [5.97–9.22]	23.62 [21.48–25.83]	100.42 [82.50–130.47]	**2.91 (0.7)**	**2.62 (± 0.2)**	**R**
	U. Dinajpur	Itahar	160	40	38	0	23.75	0	**23.75**	11.02 [9.04–12.82]	31.57 [29.03–34.46]	121.92 [99.52–159.70]	**6.06 (0.3)**	**2.80 (± 0.2)**	**R**
**0.05% deltamethrin**	Darjeeling	Siliguri	160	40	157	1	98.13	2.50	**98.08**	4.48 [2.38–5.56]	12.09 [8.84–14.35]	43.28 [36.38–63.51]	**12.93 (0.02)**	**2.97 (± 0.3)**	**S**
		Matigara	160	40	158	0	98.75	0	**98.75**	6.45 [5.26–7.46]	11.60 [10.57–12.51]	24.63 [22.25–28.27]	**0.95 (0.9)**	**5.03 (± 0.5)**	**S**
		Khoribari	160	40	160	0	100.00	0	**100**	6.69 [5.63–7.67]	13.07 [11.97–14.09]	30.84 [28.20–34.41]	**0.94 (0.9)**	**4.41 (± 0.3)**	**S**
	Jalpaiguri	Dhupguri	160	40	154	2	96.25	5.00	**96.25**	7.29 [6.24–8.24]	13.82 [12.76–14.82]	31.43 [28.84–34.87]	**1.99 (0.8)**	**4.61 (± 0.3)**	**PR**
		Malbazar	160	40	158	2	98.75	5.00	**98.68**	5.05 [3.84–6.27]	11.86 [10.47–13.13]	35.49 [31.37–41.68]	**6.4 (0.3)**	**3.46 (± 0.2)**	**S**
	U. Dinajpur	Itahar	160	40	160	0	100.00	0	**100**	5.38 [4.09–6.47]	10.14 [8.94–11.14]	22.85 [20.52–26.54]	**1.09 (0.9)**	**4.67 (± 0.5)**	**S**
**5% malathion**	Darjeeling	Siliguri	160	40	160	0	100.00	0	**100**	10.85 [8.02–12.03]	17.52 [14.65–20.53]	32.39 [29.26–44.23]	**24.35 (0.0002)**	**6.16 (± 0.4)**	**S**
		Matigara	160	36	158	2	98.75	5.56	**98.68**	10.85 [8.15–12.58]	23.79 [20.27–27.49]	65.17 [56.16–87.18]	**13.64 (0.01)**	**3.76 (± 0.2)**	**S**
		Khoribari	160	40	158	0	98.75	0	**98.75**	9.31 [7.76–10.76]	23.49 [21.67–25.36]	77.04 [66.47–92.98]	**9.73 (0.08)**	**3.19 (± 0.2)**	**S**
	Jalpaiguri	Dhupguri	160	40	160	2	100.00	5.00	**100**	13.70 [10.09–15.51]	25.31 [20.97–29.99]	55.59 [49.19–76.13]	**24.83 (0.0002)**	**4.81 (± 0.3)**	**S**
		Malbazar	160	35	158	1	98.75	2.86	**98.71**	9.75 [8.40–10.98]	20.90 [19.44–22.37]	55.64 [49.69–63.99]	**6.31 (0.3)**	**3.87 (± 0.2)**	**S**
	U. Dinajpur	Itahar	160	40	156	0	97.50	0	**97.50**	12.71 [6.77–13.47]	21.68 [15.34–28.79]	43.08 [40.94–81.72]	**65.38 (<0.0001)**	**5.52 (± 0.3)**	**S**

*T = Test, **C** = Control, **CM** = Corrected Mortality

**#S** = Susceptible (CM ≥98%); **R** = Confirmed Resistance (CM <90%); **PR** = Possible Resistance (CM = 90–97%)

**Fig 2:**
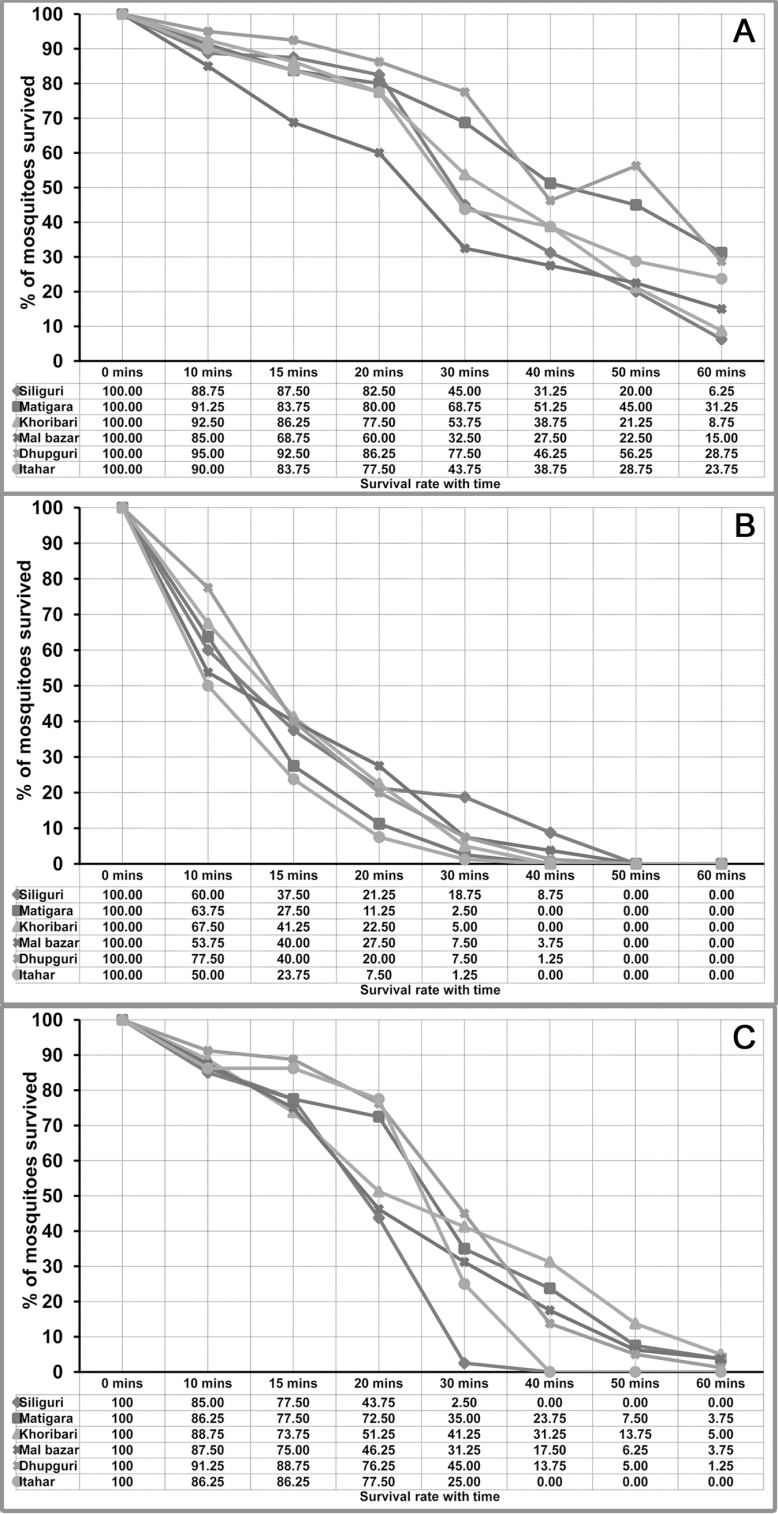
Survival rate of *Aedes albopictus* against 4% DDT (A), 0.05% deltamethrin (B), 5% malathion (C) in West Bengal.

The knock down time (KDT_10_, KDT_50_, KDT_95_) for DDT, deltamethrin, and malathion showed a linear probit for knock-down rates with time in most of the study sites (Table 3). The observed KDT_50_ values were 23.62 to 51.39 mins for DDT, 10.14 to 13.82 mins for deltamethrin, and 17.52 to 25.31 mins for malathion. The KDT_95_ values for DDT were 80.10 to 212.11 mins, for deltamethrin 22.85 to 43.28 mins and for malathion 32.39 to 77.04 mins. The survival rate of *Ae*. *albopictus* against DDT, deltamethrin, and malathion over an exposure time of 1 hour is given in Fig 2A–2C. During 1 hour of exposure, the knock down rate (KDR) varies from 68.75% - 93.75% for DDT, 100% for deltamethrin, and 95.00% - 100% for malathion.

### Detection of *kdr* mutations in *Ae*. *albopictus*

DNA was isolated from 30 dead and 10 alive, deltamethrin-exposed *Ae*. *albopictus* mosquitoes and used for PCR amplification. For detection of *kdr* mutations three DNA fragments of 480 bp, 740 bp, and 280 bp for domain II, III, and IV of *VGSC* gene were amplified, respectively. None of the six important *kdr* mutations (i.e., S953P, I975M/V, L978, V980G, F1474C, D1703Y) were found among the studied mosquitoes. We detected 3 synonymous mutations in domain II, 1 non-synonymous and 3 synonymous mutations in domain III, and 5 synonymous mutations in domain IV. The frequencies of observed mutations are presented in Table 4. The DNA sequences have been submitted to GenBank under accession nos.MF776970 and MF774494.

**Table 4:** Prevalence of SNPs in *VGSC* gene of *Aedes albopictus* in West Bengal.

Domains	SNPs		Occurrence of mutations		
	Amino acids	Codon change	N	%	95% CI
II	V942**V**	GT**G**>GT**A**	6	15.0%	7.06–29.07
	L946**L**	**C**TG>**T**TG	5	12.5%	5.46–26.11
	C947**C**	TG**T**>TG**C**	40	100%	91.24–100.00
III	D1445**D**	GA**T**>GA**C**	6	15.0%	7.06–29.07
	G1453**G**	GG**C**>GG**A**	5	12.5%	5.46–26.11
	F1468**F**	TT**C**>TT**T**	7	17.5%	8.75–31.95
	S1485**L**	T**C**G>T**T**G	3	7.5%	2.58–19.86
IV	A1691**A**	GC**T**>GC**C**	4	10.0%	3.96–23.05
	G1694**G**	GG**G**>GG**C**	3	7.5%	2.58–19.86
	D1709**D**	GA**C**>GA**T**	5	12.5%	5.46–26.11
	N1712**N**	AA**T**>AA**C**	6	15.0%	7.06–29.07
	F1713**F**	TT**T**>TT**C**	8	20.0%	10.5–34.76

## Discussion

Emergence and spread of insecticide resistance is the biggest challenge to control vector-borne disease transmission [38]. In *Aedes* mosquitoes there are two major mechanisms for pyrethroid resistance: increased detoxification and mutation in the *VGSC* gene. To date more than 50 different *VGSC* mutations have been identified in different insect species [19]. The six non-synonymous amino acid substitutions: S989P, I1011M, L1014F, V1016G in domain II, F1534C in domain III and D1763Y in domain IV of house fly are found to be associated with pyrethroid resistance. These codons are orthologous to the codons 953, 975, 978, 980, 1474 and 1703, respectively of *Ae*. *albopictus*. The involvement of other mutations in pyrethroid resistance remains to be investigated. The L1014F, at S6 subunit of domain II was the first pyrethroid-resistance-associated mutation identified in the house fly and German cockroach [39, 40, 41]. I1011M was identified in domestic house fly from Brazil, Guyana, whereas V1016G was identified from Indonesia and Thailand [42]. Later, different substitutions, I1011V and V1016I, were found in *Ae*. *aegypti* populations from Latin America [43]. The most significant F1534C, located in S6 subunit of domain III was discovered in DDT/permethrin-resistant *Ae*. *aegypti* in Thailand and Vietnam [44, 45]. The adult insecticide susceptibility bioassay is applied to determine the lethal dose of different insecticides by direct exposure. Additional tests, such as polymorphisms in marker genes and biochemical assays of different enzymes are used as supplementary evidence to clarify the results of bioassays and potential mechanisms.

In the present study, we determined the susceptibility status of *Ae*. *albopictus* against DDT, deltamethrin, and malathion. The results showed that *Ae*. *albopictus* is significantly resistant to DDT with a higher KDT and KDR and a lower mortality rate. Similar observations have also been reported from other parts of the country [11, 12, 14]. Though DDT is not in use against *Aedes* vector mosquitoes, this compound is still in use for control of malaria vectors. The present study areas have been highly endemic for malaria for a long time, with the exception of Itahar. Thus, *Aedes* mosquitoes have been exposed to DDT for many generations which might be the cause of the high level of resistance that has developed to DDT. *Ae*. *albopictus* from the present study areas were susceptible to deltamethrin and malathion. Pyrethroid resistance in adult *Aedes* sp. is a problem worldwide. The level of resistance varies from region to region. A lower level of resistance is found in Asian, African, and Northern American countries, [46, 47, 48, 49] whereas higher levels of resistance are found in South American countries [50, 51]. In the present study, lower values of knock down time and knock down rate were observed in *Ae*. *albopictus* against deltamethrin and malathion. The KDT values recorded in the present study did not follow a normal distribution pattern which indicates that the prevailing *Ae*. *albopictus* population is susceptible to these insecticides.

In India, temephos is used as larvicidal agent. In contrast to adult susceptibility, higher levels of larval resistance have been found in Asian, African, and North American countries [49, 52, 53, 54, 55]. In the present study we found that the *Ae*. *albopictus* larvae were sensitive to temephos in Khoribari and Dhupguri (RR_99_<3); showed a low level of resistance in Matigara, Malbazar, and Itahar (3<RR_99_>5), and moderate resistance in Siliguri (5<RR_99_>10) [33].A similar type of observation was also reported from the north eastern part of India [11, 12]. The Siliguri Municipal Corporation is the only urban site in the present study, where temephos has been in use for a long time. A longer duration of exposure to temephos might be the cause of the observed moderate level of resistance against it in *Ae. albopictus* from Siliguri. In contrast, a recent report from the northern part of West Bengal showed susceptibility of *Ae*. *albopictus* larvae to temephos assessed by larval susceptibility and bioassay of detoxifying enzymes [56].

The KDR is a mechanism of DDT and pyrethroid resistance. Mutations at codons 953, 975, 978, 980, 1474, 1703 of the *VGSC* gene of *Ae*. *albopictus* have been found to be associated with reduced susceptibility to both DDT and pyrethroids [22, 23]. As per our present study, the only previous report from India did not reveal any mutation in the *VGSC* gene of *Ae*. *albopictus* [8] but two other reports reveal mutations at codon F1534C [57] and at codon T1520I + F1534C of the *Ae*. *aegypti VGSC* gene [13]. In the present study, we detected only one non-synonymous mutation at S1485L in three samples. Interestingly, all three mosquitoes were susceptible to deltamethrin. So, the role of this mutation in pyrethroid resistance cannot be explained. We also detected 11 synonymous mutations among both dead as well as live deltamethrin-exposed mosquitoes.

We did not assess the detoxifying enzyme levels associated with DDT and deltamethrin resistance. The higher susceptibility level in deltamethrin with absence of important *kdr* mutations and higher susceptibility to malathion indicate that these two insecticides are still effective in the study areas. The susceptibility status of temephos as a larvicide should be monitored closely as moderate and lower levels of resistance were observed in mosquitoes from a few study sites. A similar study is highly recommended for monitoring and early detection of pyrethroid and malathion resistance in other parts of the country.
